# Translation of the Amsterdam Preoperative Anxiety and Information Score (APAIS) into the Amharic Version and Its Validation for Evaluation of Preoperative Anxiety

**DOI:** 10.4314/ejhs.v31i2.18

**Published:** 2021-03

**Authors:** Blen Ayele, Mahelet Tadesse, Rahel Tilahun, Berhanu Nega

**Affiliations:** 1 Department of anesthesiology, school of medicine, Addis Ababa University, Addis Ababa, Ethiopia; 2 Department of surgery, school of medicine, Addis Ababa University, Addis Ababa, Ethiopia

**Keywords:** Forward/backward translation, APAIS, preoperative anxiety, Cronbach's, alpha

## Abstract

**Background:**

Peri-operative anxiety is a vague, unpleasant feeling, the source of which is often nonspecific and unknown to the individual. It affects all aspects of anesthesia. Although the magnitude and consequences of preoperative anxiety are well documented in the developed world, there are limited studies conducted in Ethiopia. The primary aim of this study is to produce the Amharic version of APAIS and evaluate its validity in assessing the prevalence of preoperative anxiety in surgical patients.

**Methods:**

A cross-cultural adaptation process using a forward/backward translation of the APAIS scale was performed. The Amharic version was then tested in 365 sampled elective adult surgical patients scheduled for surgery at Tikur Anbessa specialized Hospital. The validity of the translated version was also checked by evaluating its psychometric properties of internal validity and acceptability.

**Result:**

The results showed that the reliability of the APAIS-Amharic was high (Cronbach's alpha of 0.87) and the data collected was a good fit (RMSEA of 0.04). In addition, the questionnaire was well-accepted 100% with no missing values for each dimension of the APAIS. The mean APAIS scores for total anxiety and desire for information were 11.6 and 6.0 respectively and 58.9% of the study participants had anxiety and those patients, who had some form of formal education, came from urban areas, had previous anesthesia experience and complications and who had average or high information requirement was more likely to be anxious.

**Conclusion:**

APAIS-Amharic is a reliable and acceptable tool for measuring patients' preoperative anxiety and their need for information. It can be used routinely as a screening instrument at pre-anesthesia clinics to assess patients' level of anxiety.

## Introduction

Perioperative anxiety is a vague, uneasy feeling, the source of which is often nonspecific and unknown to the individual. It is the subjectively unpleasant feeling such as the feeling of imminent death (1). It has the potential to affect all aspects of anesthesia during preoperative, induction, post-operative and recovery periods (3,4). According to Woldegerima et al, preoperative anxiety was observed in 59.6% of patients. Urban residents, low income, young age, disability, dependency, fear of death, and concern about well being of their family were found to have association with preoperative anxiety. (5)

Preoperative anxiety level is difficult to measure accurately. To measure preoperative anxiety, several validated questionnaires are currently available like Amsterdam Preoperative Anxiety Information Scale (APAIS), State-Trait Anxiety Inventory (STAI), Hospital Anxiety and Depression Scale (HADS), Visual Analogue Scale (VAS), Multiple Affect Adjective Checklist (MAACL) and newly developed specific instruments like the Anxiety Specific to Surgery Questionnaire (ASSQ) (6,7). In 1996 the Dutch group of Moerman developed the Amsterdam Preoperative Anxiety and Information Scale (APAIS) (10). This questionnaire consists of six items and is, therefore, an economical instrument. Many studies used the State-Anxiety-Scale (STAI) for evaluation of anxiety. The APAIS correlates with the STAI with r=0.74, r=0.67 as well as r=0.64, which is a good indicator of its validity (3,24). Furthermore, the APAIS was applied and proved helpful in several international studies. The APAIS has also been validated in surgical patients, whereas the STAI scale was validated in the general population (8). Unlike APAIS, the use of anxiety screening instruments like STAI usually takes a longer time. Therefore, using the APAIS that has only six items has become the standard of practice for the evaluation of perioperative anxiety in many countries. Countries like Japan, Mexico, Italy, Turkey, Thailand and South Korea have developed the APAIS version of their language and tested its validity with excellent outcomes (3).

Ethiopia is a multi-linguistic country, and Amharic is currently used as a federal language of the country. Literature review showed that APAIS has neither been previously translated into Amharic nor used for assessment of preoperative anxiety. The primary objective of this study was to translate the APAIS into Amharic language and evaluate its validity. The secondary endpoint was to determine the prevalence of preoperative anxiety in Tikur Anbessa Specialized Hospital (TASH) and the factors associated with it.

## Materials and Methods

**Figure 1:**
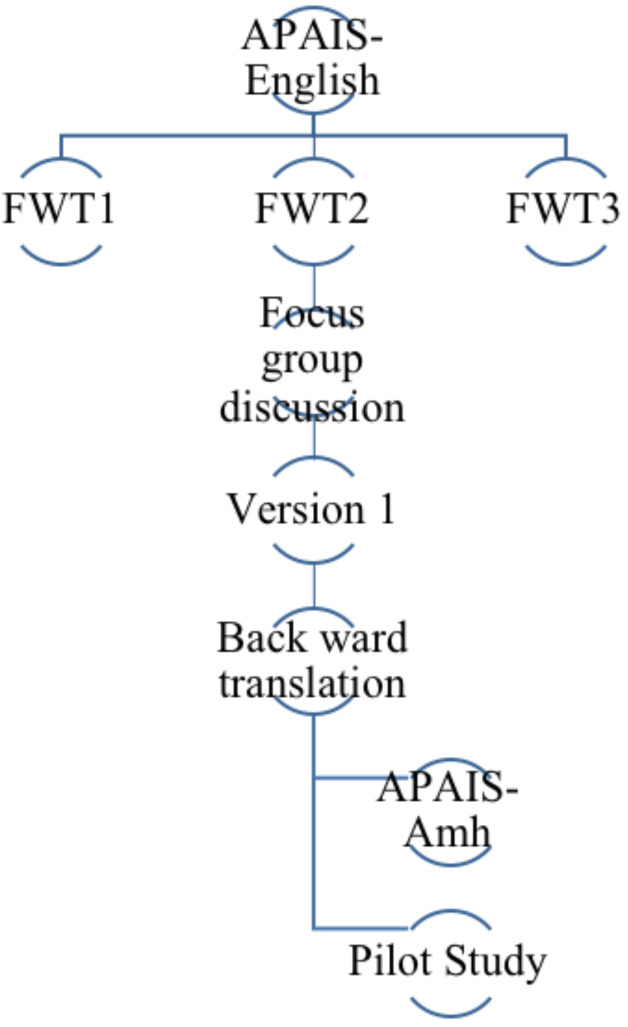
Process of translation and cross-cultural adaptation of APAIS English to APAIS-Amharic

**Design:** A cross-cultural adaptation process using a forward/backward translation of the APAIS scale was performed to produce Amharic version of the APAIS scale (APAIS-Amharic). A psychometric validation was subsequently done for the adaptation of APAIS-Amharic scale (Figure 1).

**Questionnaire of the source language**: We used APAIS as a source language for translation. The questionnaire is a self-reported six-item questionnaire developed by Nelly that was used for the assessment of pre-operative anxiety. Anxiety related to anesthesia is assessed by two questions. Anxiety related to surgery and need for two also assesses information questions each. Each question is rated with a five point Likert scale (ranging from 1 ‘not at all’ to 5 ‘extremely’).

**Process of translation and Cross-cultural adaptation:** Three independent professionally trained bilingual expert translators, who are familiar with the culture of both languages were recruited. They conducted the forward translation and developed three separate Amharic versions of the questionnaire. Subsequently, in order to reconcile and synthesize the forward translation, a focus group discussion was carried out on the three versions. The focus group members were all bi-lingual and composed of three anesthesiologists, one surgeon and one English language teacher. The objective of the focus group discussion was to agree and produce a combined and culturally adapted version, which is conceptually equivalent to the original source language (Version 1). In order to resolve discordance, a blinded professional translator who had no access to the original source language did using Version 1 of the translation a backward translation. It was then compared with the original APAIS. The final version of APAIS-Amharic was then developed and used for our pilot study.

**Pilot testing:** This pilot study was done with the aim to ascertain the Amharic version of APAIS conceptual meaning correctness and the ease of administration of the tool. Ethical approval was obtained from Addis Ababa University (AAU) College of health science IRB board.

**Design:** Institution based prospective cross-sectional study

**Setting:** The study was conducted in TASH, Addis Ababa, Ethiopia. TASH is Ethiopia's largest general public hospital providing a tertiary level referral treatment. It also serves as a primary teaching hospital for both clinical and preclinical disciplines. The study was conducted on elective adult surgical patients from the 1^st^ of July to August 30, 2019.

**Study subject:** The study population was adult patients of age > 18 years admitted to the hospital for elective surgeries with ASA I-III. All the study subjects spoke and understood Amharic very well and gave consent to participate in the study. Emergency patients and patients who were not willing to give consent, patients with mental retardation, dementia, or psychiatric disorders and patients on premedication were excluded from the study.

The sample size was determined by using a single proportion for a finite population with the assumption of 95% confidence interval, 5% margin of error. A prevalence of 61% (29) was used and the calculated sample size is found to be 365. All consecutive patients that fulfill the inclusion criteria were included in the study.

**Data collection and psychometric evaluation**: The psychometric properties of the Amharic version were evaluated after collecting data using the translated version. Interviews with the patients were performed a night before surgery during the preoperative examination and before any premedication was given. Trained BSC nurses who were blind to the study carried out the interview. Two supervisors observed the process of data collection and also checked the consistency and cleanliness of the data daily.

The APAIS-Amharic comprises six statements. All statements were scored on a 1 to 5 Likert scale. (1-Not at all, 5- Extremely) The scores from questions 1 and 2 are added to show anesthesia-related anxiety, 4 and 5 are added together to show the patients' surgery-related anxiety while the sum of scores from questions 1, 2, 4 and 5 show the patient's total level of anxiety. In addition, scores for questions 3 and 6 are added together to identify the patent's need for information. A patient with a score of ≥11 on the anxiety scale experiences anxiety. On the information scale, patients scoring 2–4 are classified as having little or no information requirements, 5–7 as having an average information requirement and 8–10 as having high information requirements. Patients were also asked about the cause of their anxiety regarding the anesthesia if there were any. They could select more than one cause if it is the case.

**Statistical analysis:** It was made using SPSS version 24. Descriptive analysis was performed to describe the number and percentage of sociodemographic characteristics and other variables. To evaluate the correlation between variables, chi-square test of independence correlation analysis using p-value was used. The results are then presented in text, tables, and graphs based on the types of data. The psychometric evaluation of the national scaling system was done by checking its internal validity and acceptability.

**Internal validity:** This included checking for reliability and adequacy of the Amharic version model of the APAIS. The reliability of the internal consistency of each dimension was assessed using Cronbach's alpha coefficient. Cronbach's alpha is a function of the number of items in a test, the average covariance between item pairs and the variance of the total score. This means measuring how closely related items are as a group. For this study, Cronbach's alpha coefficient higher than 0.7 was expected for each scale.

The adequacy of the model was also explored using a global index that is responsive to sample size and complexity: the root mean square error of approximation (RMSEA) that measures how well the data collected fits the model. An RMSEA lower than 0.08 indicates a fair fit, less than 0.06 acceptable fit and less than 0.05 a good fit.

**Acceptability:** The percentage of missing answers was also used to explore the acceptability of the Amharic version among the patients. However, to ensure data quality, the validation analysis was not performed on records with more than 25% of the responses missing. In addition, the findings of the study were compared with other similar studies done using the state-trait anxiety inventory questionnaire. The final Amharic version of APAIS and its five point Likert scale are shown in Tables 1 and 2.

**Table 1:** Items of the Amsterdam Preoperative Anxiety and Information Scale English and Amharic version

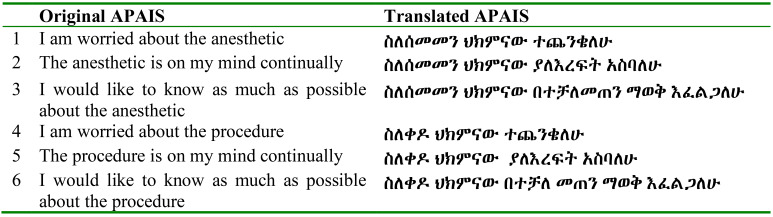

**Table 2:** Likert scale ranges English and Amharic version

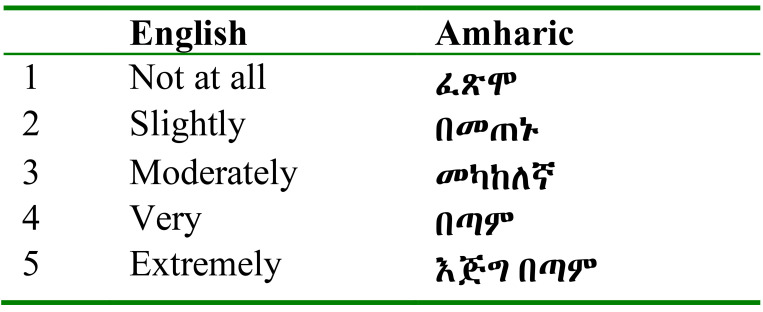

## Results

**Socio-demographic characteristics and item scores:** A total of 365 patients were incorporated into this study with a 100% response rate. The study enrolled 200(54.8%) males, and 53% of the participants (194) were in the age range of 18–49. Concerning the educational status of the participants, 32.9% (120) were illiterate. Out of the total sampled patients, 61.9% (226) were urban residents.

**Current surgery and previous experience**: Around half of the study participants were admitted for urologic (30.7%) and general surgeries (17.5%), followed by orthopedic (17%), obstetrics (9.6%) surgery, cardiothoracic (9.5%), neurologic (7.1%), ENT (5.7%) and gynecologic (3.4%) surgeries.

One-third of these patients had the experience of major surgery previously (33.7%) while the rest did not have any previous major operation and anesthesia experience. Out of those patients who had surgery experience, the majority (66.7%) had general anesthesia experience and 30.1% encountered surgery or anesthesia-related complications in their previous surgeries.

Out of those patients who reported the presence of either anesthesia or surgery-related complication in their previous surgeries, 40.5% had an infection as a complication followed by delayed recovery (29.7%) and others (19%), which included, pain and incomplete cure.

**Results of the APAIS:** The mean APAIS scores were as follows: anxiety for anesthesia (5.9 ± 2.6); anxiety for surgery (5.6 ± 2.4); total anxiety score (11.6 ± 4.6) and desire for information (6.0 ± 2.4) (Table 3). In addition, 58.9% of the study participants had anxiety on the APIS score, scoring 11 or more while 41.1% scored in the range of 2–11, hence no anxiety. Around three fourth of the participants had either average (37.5%) or high (35.9%) information requirement regarding the anesthesia and surgery, while only 26.6% of the participants had little or no information requirement.

**Table 3:** Results of the APAIS score, mean standard deviations and Cronbach's Alpha of anxiety scores and need for information scores, in TASH, Addis Ababa, Ethiopia, July Aug 2019. (N- 365)

	N	Min Score	Max Score	Mean	SD	Cronbach's Alpha
Anesthesia related anxiety	365	2	10	5.97	2.65	0.85
Surgery related anxiety	365	2	10	5.60	2.45	0.84
Total anxiety Score	365	4	20	11.59	4.63	0.87
Total need for Information score	365	4	20	6.05	2.37	0.75

**Figure 2:**
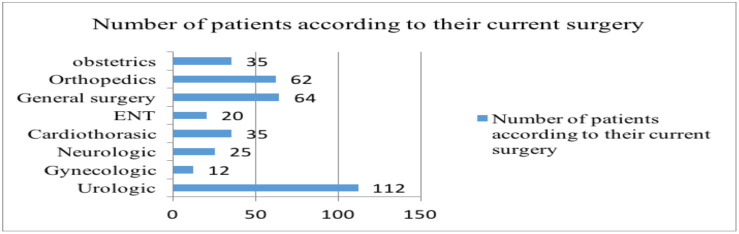
Causes of anesthesia related worry of elective surgical patients in TASH, Addis Ababa, Ethiopia, July-August 2019 (N-312).

Out of the total interviewed 365 elective surgical patients, 312 reported at least a slight worry about the anesthesia on the first question of the APAIS. Further, these patients were asked about the reason for their worry. The unknown cause was answered by 22.4 % of them, postoperative pain by 21.5%, pain during surgery by 20.5% and permanent disability by 13.8% as the cause of their worry (Figure 2).

**Predictors of preoperative anxiety:** To evaluate the correlation between sociodemographic characteristics and anxiety score as well as the need for information and anxiety score, chi-square test of independence was performed to calculate p-value as seen in Table 4. The proportion of patients who had anxiety (scored 11 or more on the APAIS) did not differ by age and sex [X^2^ (1, N=365) = 8.9, p>.05]. However, according to this study, it was found that those patients who had some form of formal education came from urban areas, had previous anesthesia experience and had previous anesthesia-related complications were more likely to be anxious (P < 0.05).

**Table 4:** The relationship of APAIS scores with patient characteristics and their need of Information, in TASH, Addis Ababa, Ethiopia. July-Aug 2019. (N - 365)

		Total Anxiety Score			
				
		No anxiety (2–11)	Anxiety (>=11)	Total	P-value
Age -	18–59	128	180	308	0.67
	>=60	22	35	57	
Sex	Male	82	118	200	0.96
	Female	68	97	165	
Educational status	No education	62	58	120	0.004
	Some education	88	157	245	
Residence	Urban	79	147	226	0.002
	Rural	71	68	139	
Previous anesthesia experience	Yes	39	84	123	0.009
	No	111	131	242	
Previous Anesthesia or Surgery Related Complication	Yes	7	30	37	0.04
	No	32	54	86	
Information requirement	No/little	49	48	97	0.03
	Average/high	101	167	268	

Those who have some formal education were more likely to experience pre-operative anxiety as compared to those who do not have any education. Patients who came from urban area were more likely to be anxious than those who came from rural area. Patients with previous exposure to anesthesia who had complication were more likely to develop preoperative anxiety as compared to those who had no previous exposure and complication related to the anesthesia. In addition, while examining the relationship between patients' need for information and their anxiety score, it was found that there is a significant relationship between these variables. Those patients who had no or little information requirement regarding surgery and anesthesia were less anxious than those who had an average or high information requirement (P < 0.05) (Table 4).

**Validation**

**Content validity:** During the focused group discussion, the panel of the five experts reviewed the translation done by the three independent professionally trained bilingual expert translators. The panel verified the conceptual equivalence of this version and decided to replace 
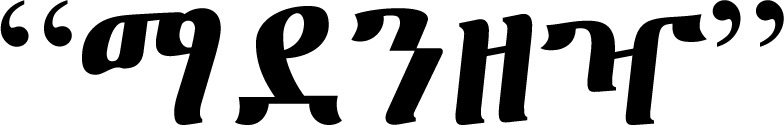
 with 
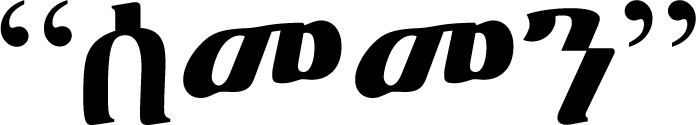
 (Items 1, 2 and 3), and 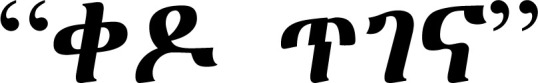 with 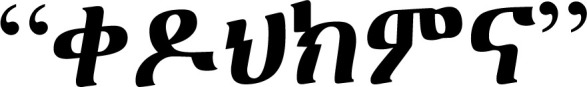 (Items 4, 5 and 6) a much more common term used by Surgical Society of Ethiopia and Ethiopian Society of Anesthesiology.

**Internal validity:** The internal consistency reliability of the Amharic version of the APAIS was high, i.e., the Cronbach's alpha values ranged from 0.75 to 0.85. This shows an acceptable internal consistency of the desire for information scale and good internal consistency of anesthesia-related anxiety, surgical related anxiety, and global anxiety scores. In addition, the confirmatory factor analysis indicated a good fit with the root mean square error of approximation of 0.04 (<.05).

**Acceptability:** From the collected data, it was found that there were no missing values for each dimension of the APAIS (100% acceptability). These results indicate that the questionnaire was well accepted.

## Discussion

The APAIS has been initially designed and validated in Dutch, the construct validity was evaluated by factor analysis and external validity has also been performed. The two scales, anxiety and information need, assess important constructs for anesthesia and surgery. In this study, it was found that the reliability of the Amharic version of the APAIS was high as the internal consistency determined by the Cronbach's alpha was 0.75 for the total need for information score and 0.87 for total anxiety score (Cronbach's alpha coefficients higher than 0.7 shows acceptable internal consistency while Cronbach's alpha coefficient higher than 0.8 shows good internal consistency.) This result is similar to other studies done in French and Srilanka where Cronbach's alpha was 0.86 for the Sinhala version and ranged from 0.76 to 0.84 for the French version (9,31).

In addition, the data collected fits the Amharic version of the APAIS well as it is shown by the RMSEA of 0.04, which is a good fit (<0.05). This result, when compared to the French version of the APAIS with an RMSEA result of 0.06 shows that the Amharic version has a better fit. Furthermore, the acceptability of the APAIS-Amharic was 100% as there were no missing values for each of its dimensions. Therefore, it can be used as an effective tool to measure preoperative anxiety levels in Amharic speaking patients. In this study, the overall prevalence of preoperative anxiety was 58.9% when the APAIS score was 11 and more. This result is consistent with two recent studies done in Ethiopia using the gold standard STAI scale i.e., in Gondar University Hospital (59.6%) and Debre Markos and Felege Hiwot hospitals (61%) (5,28,32). This result was found to be higher than another study conducted in Nigeria where the prevalence of anxiety was 51%. However, the prevalence in our study was lower than the previous study conducted in Sri Lanka using a similar tool in which the overall prevalence of preoperative anxiety was 76.7%. This could be attributed to the socio-cultural differences of the two populations where our society may not have the culture to openly report about their worry regarding anesthesia and surgery out of their respect for surgeons and anesthesiologists.

In addition, the mean APAIS score for anesthesia anxiety and surgery anxiety was similar with the means of 5.9 ± 2.6 and 5.6 ± 2.4 respectively unlike the results of the study done in Pakistan where patients feared surgery significantly more than anesthesia (25). The total mean anxiety score was 11.6 ± 4.6, and the mean desire for information score was 6.0 ± 2.4 which was higher compared to the mean APAIS score for global anxiety (7.2 ± 3.7) and mean desire for information (5.7 ± 2.3) of that of the French version. This might be explained by the fact that enough time may not be given for providing preanesthesia and surgery information in our setup and hence a higher mean score of anxiety. Furthermore, out of those patients who at least reported a slight worry about the anesthesia, the top three reasons (in descending order) responsible for their anesthesia-related worry were found to be unknown cause, post-operative pain and pain during surgery. This is a different result from that of the Srilankan study where awareness during anesthesia was the number one cause for anxiety. However, the most common causes of preoperative anxiety in other studies included concern about family, fear of complication, fear of death and fear of postoperative pain (25,26,28,29). Therefore, for anesthesiologists, it would be important to address these causes of anesthesia-related worry of patients during their pre-anesthetic evaluation in order to alleviate their anxiety.

Different factors can affect anxiety levels in patients, which vary from country to country. In this study, the socio-demographic characteristics that were found to be significantly associated with preoperative anxiety were educational status and residence. However, our study showed lack of significant effect of age and gender on the level of preoperative anxiety. Those patients who had some form of formal education and came from urban areas were found to be more anxious than those who are illiterate and came from rural areas. Highly educated people who have a tendency to extrovert their feelings, information-seeking behavior, and awareness of possible complications can explain this association. Also, as they get incomplete information from different sources, their anxiety level will escalate (5). This finding is similar with a study done in Jimma, Ethiopia, (using STAI scale) and Pakistan (using VAS). The level of preoperative anxiety appeared to increase with increasing level of education and opposite to the finding of the northwest part of Ethiopia (using STAI scale) where the level of anxiety decreases with increasing level of education (25,28,29). In the current study, it was also found that patients who had previous surgery or anesthesia experience and had previous anesthesia-related complications were more likely to be anxious than those who did not With the presence of previous complications, patients are more obviously become anxious because of the fear of complications happening in their current surgery as well. How ever, even in the absence of complications, patients might have experienced stressful events like the death of their neighboring patients during their last admission, which can increase their level of anxiety on their current surgery (31).

This finding is, however, different from findings of other studies that show more experienced patients were less anxious (24,29). This study also showed a high positive correlation between high information seekers and anxiety scores. Those patients who had average or high information requirements were more anxious than those who had no or little information requirement regarding anesthesia and surgery. This finding is in line with other studies where patients with extremely high information requirement were anxious patients. Further, it was suggested that patients with a monitoring coping style become anxious when they are not provided with as much information as they need. However, it is also important to realize that extensive information is not always useful and may even induce anxiety particularly in patients with a blunting coping style (10,24). Therefore, anxious patients may benefit from more information based on their information requirements.

Finally, although this study showed that the Amharic version of the APAIS is a reliable and acceptable tool in terms of internal validity, other domains of psychometric evaluations like differential item functioning and external validity were not done. In addition, this study did not look into the preoperative anxiety in pediatrics and emergency patients. Furthermore, the level of anxiety at pre-anesthesia clinics was not compared with that of the night before surgery, and anxious patients were not followed during and after surgery to evaluate their outcomes.

In conclusion, the Amharic version of the APAIS is a reliable and acceptable tool for measuring patients' preoperative anxiety for Amharic speaking patients. It can be used for Amharic speaking patients as a screening instrument at pre-anesthesia clinics to assess patients' levels of anxiety and hence provide a platform to clarify their doubts about anesthesia and plan for the appropriate premedications. The study also showed that the prevalence of anxiety was high and the level of anxiety was significantly associated with educational status, residence, previous surgery experience, and information requirement. Therefore, the use of the Amharic version of the APAIS for routine anxiety and the need for information assessment should be given due consideration for Amharic speaking patients. We also recommend for appropriate information to be given for patients in accordance to their need for information at pre-anesthesia clinics before surgery. Allocating enough time and providing appropriate pre-operative information for surgical patients will accordingly address their causes of worry.
